# Diagnosis and management of retroperitoneal ancient schwannomas

**DOI:** 10.1186/1477-7819-7-12

**Published:** 2009-02-02

**Authors:** Haroon A Choudry, Mehrdad Nikfarjam, John J Liang, Eric T Kimchi, Robert Conter, Niraj J Gusani, Kevin F Staveley-O'Carroll

**Affiliations:** 1 Department of Surgery, Penn State Milton S. Hershey Medical Center, Hershey, Pennsylvania, USA; 2 Department of Surgery, University Hospitals, Case Medical Center, Cleveland, Ohio, USA; 3 Department of Pathology, Penn State Milton S. Hershey Medical Center, Hershey, Pennsylvania, USA

## Abstract

**Background:**

Ancient schwannomas are degenerate peripheral nerve sheath tumors that very rarely occur in the retroperitoneum. They generally reach large proportions before producing symptoms due to mass effect. We describe three cases of retroperitoneal ancient schwannomas and discuss the diagnosis and management of these tumors.

**Case presentations:**

Three female patients with retroperitoneal ancient schwannomas were reviewed. One patient presented with several weeks of upper abdominal pain and lower chest discomfort, whereas back pain and leg pain with associated weakness were predominant symptoms in the remaining two. Abdominal imaging findings demonstrated heterogeneous masses in the retroperitoneum with demarcated margins, concerning for malignancy. The patients successfully had radical excision of their tumors. Histological examination showed encapsulated tumors that displayed alternating areas of dense cellularity and areas of myxoid matrix consistent with a diagnosis of ancient schwannoma.

**Conclusion:**

A diagnosis of ancient schwannoma should be entertained for any heterogeneous, well encapsulated mass in the retroperitoneum. In these cases less radical surgical resection should be considered as malignant transformation of these tumors is extremely rare and recurrence is uncommon following excision.

## Background

Ancient schwannomas are a rare variant of peripheral nerve sheath tumors, or schwannomas. The term "Ancient" refers to the histological degenerative features, which are acquired with increasing age in these tumors. Nuclear atypia is a common feature and often leads to the erroneous diagnosis of malignancy. They are slow growing and may produce vague local symptoms, but are usually diagnosed incidentally. They are an uncommon cause of a retroperitoneal mass, and are classically encapsulated, highly vascular and have a distinctive radiological appearance [1,2]. Malignant transformation is extremely rare and recurrences are uncommon following surgical resection.

We describe three cases of retroperitoneal ancient schwannomas managed surgically at our institution over the last two years.

## Case presentations

### Case 1

**Figure 1:**
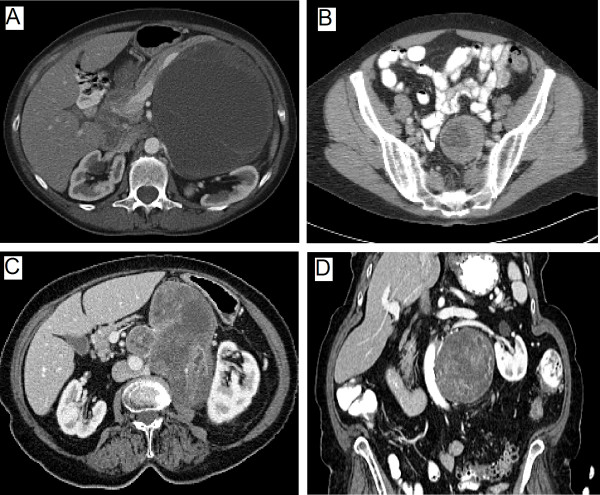
**Computed tomography findings of three retroperitoneal ancient schwannomas**. A. Large partly cystic and solid tumor displacing the pancreas and splenic vein anteriorly and compressing left kidney. Tumor concerning for a sarcoma or pancreatic neoplasm. B. Well defined tumor extending from the retroperitoneum into left pelvis adjacent to sigmoid colon. Region of vascular enhancement around tumor periphery can clearly be seen. C&D. Large heterogeneous enhancing mass adjacent to the aorta and left kidney shown in transverse and sagittal sections. Partial encasement of the aorta and left renal artery is demonstrated concerning for a malignancy.

**Figure 2:**
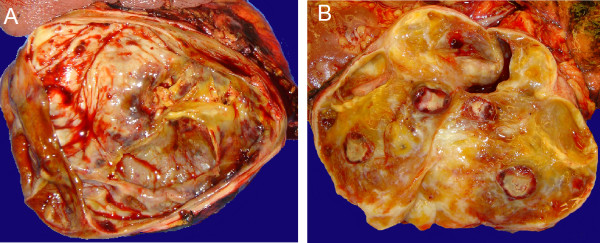
**A. Macroscopic section of large tumor displacing pancreas showing large cystic regions, areas of hemorrhage and calcification B. Tumor showing fibrotic, calcified and cystic regions**.

**Figure 3:**
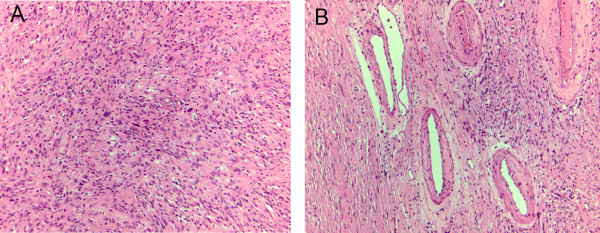
**A. The tumor is composed of spindle cell proliferation with hyper-and hypocellular areas and focal cystic degeneration**. Rare atypical large nuclei are present in the absence of significant mitotic activity B. Hyalinized and thickened blood vessels can be observed and fibrotic stroma. Immuno-staining was positive for S-100 (not shown).

A 60 year-old female was evaluated in the emergency room with a two week history of chest and epigastric pain. A cardiac work-up and upper and lower gastrointestinal endoscopy was unrevealing, however, a computed tomography (CT) scan of the chest, abdomen and pelvis showed a large, well circumscribed, septated cystic lesion with a few scattered calcifications adjacent to the pancreas, measuring 12.0 × 13.0 × 12.0 cm, without any signs of metastases. The epicenter of the mass appeared to be the superior retroperitoneal region with displacement of the tail of the pancreas, kidney and stomach (Fig 1A). A presumptive diagnosis of a retroperitoneal liposarcoma or possible pancreatic neoplasm was entertained. Tumor markers performed, including carcinoembryonic antigen (CEA) and carbohydrate antigen 19.9 (CA 19.9) were not elevated. The patient underwent a radical excision of the mass with *en bloc *resection of the distal pancreas, spleen and left adrenal gland. This mass had solid and cystic features (Fig 2A). Histopathology revealed an ancient schwannoma (Fig 3). All margins were clear and the patient was well at 3 months follow-up.

### Case 2

A 71 year old female with 8 months of back and leg pain. The pain was of a slow onset and initially thought related to degenerative spinal changes based on plain x-rays. The patient subsequently developed associated leg weakness and underwent magnetic retrograde imaging (MRI). This led to the discovery of a complex cystic pelvic mass, that was further characterized on CT of the pelvis (Fig. 1B). A well-defined 4.9 × 5.4 cm mass within the left hemi-pelvis, with a hypodense center was noted. It had well preserved peri-lesional fat planes, with no infiltration of the surrounding fat and no lymphadenopathy. Further work-up including positron emission tomography (PET) showed increased tracer uptake within the periphery of the mass concerning for a malignant process. There was no evidence of colon pathology on colonoscopy and pelvic ultrasound did not revealed any gynecologic pathology. The mass was thought to be a possible sarcoma and the patient underwent radical excision of the mass with *en-bloc *low-anterior resection without complications. Pathology revealed an ancient schwannoma with spindle cells, cystic degeneration, atypical cells, and S-100 positive staining (Fig 3). At 18 months follow-up there was no evidence of tumor recurrence.

### Case 3

An 82 year old female had a one year history of chronic lower back pain and left lower extremity weakness managed with analgesics and physical therapy. A CT scan of the spine showed evidence of degenerative changes with a mass in the retroperitoneum adjacent to the lumber spine. A CT of the abdomen and pelvis was then performed and revealed a 12 × 8.5 × 7.5 cm retroperitoneal soft tissue mass containing mixed solid and cystic components and scattered calcifications, with compression of the abdominal aorta, left kidney, posterior stomach and encasement of the left renal artery (Fig 1C &1D). A diagnostic percutaneous biopsy referred to our service for management of a newly diagnosed retroperitoneal schwannoma, with features suggestive of malignancy on percutaneous biopsy. A pre-operative course of external beam radiotherapy was given for 6 weeks with the aim of tumor size reduction, but there was no objective response. The patient underwent an *en bloc *resection of the mass with the left hemi-colon, left kidney and adrenal and a partial gastrectomy. The gross pathology specimen is shown (Fig 2B). Histopathology confirmed the diagnosis of an ancient schwannoma. Rare mitoses were seen, however, there were no overt features of malignancy. All margins were negative and the patient was free recurrence at 3 months follow-up.

## Discussion

Schwannomas (neurilemmomas) are benign soft tissue neurogenic tumors that arise from Schwann cells of peripheral nerve sheaths. The usually arise from sensory nerves, however, motor nerve origin is also reported. They most commonly manifest in the head and neck region and in the extremities [3]. Retroperitoneal schwannomas are rare and account for 0.7% to 2.7% of these tumors [4]. They classically have a slow, protracted clinical course prior to detection, and malignant transformation is uncommon. Histologically, they are encapsulated and display alternating areas of dense cellularity termed Antoni-A (AA) regions, and areas of myxoid matrix termed Antoni-B (AB) regions. AA regions are characterized by dense aggregation of spindle shaped cells arranged in parallel configurations, palisades or whorls. AB regions manifest as hypocellularity with predominantly loose myxoid matrix [3]. Immunohistochemical staining is typically positive for S-100, Vimentin and Neuron-specific enolase. Staining for smooth muscle actin (SMA) and CD117 is negative. Imaging characteristics of a schwannoma on CT is that of a well-defined, homogeneous mass with rim enhancement of the fibrous capsule following intra-venous contrast administration [5].

Ancient schwannomas are a rare variant of schwannomas, originally described by Ackerman and Taylor in 1951 [6]. They account for 0.8% of soft tissue tumors. They are characterized by distinctive degenerative tumor features including cystic necrosis, stromal edema, xanthomatous change, fibrosis, perivascular hyalinization, calcification and degenerative nuclei with pleomorphism, lobulation and hyperchromasia [7-9]. These degenerative features are attributed to the growth and "aging" of the tumor, hence the term "Ancient schwannoma." Growth of the tumor over time leads to vascular insufficiency, with resulting areas of tumor degeneration. Previous studies have correlated tumor size with progressive degenerative features [10]. Despite these degenerative changes, ancient schwannomas behave similarly to their conventional counterparts. They are benign, slow-growing tumors with rare malignant transformation [11-13].

There is a tendency to confuse ancient schwannomas with malignant tumors on imaging and histology [14]. On cytology and histology these tumors have degenerative features, including nuclear atypia and hyperchromasia. These features were noted in all the cases in our series. Confusion with malignancy can be avoided by recognizing benign features such as absence of mitosis and preservation of spindle shape with large cohesive aggregates of cells [7]. Flow cytometry assessing DNA ploidy may also help differentiate benign from malignant lesions [15]. Ancient schwannomas are predominantly found in elderly patients and manifest as deeply located, soft tissue masses in the head and neck region [16], thorax [17], retroperitoneum and pelvis [2,18] and extremities [19]. They grow slowly over years and clinical presentation may include local pressure symptoms of pain, numbness, paresthesias or they may be found incidentally.

On histology, ancient schwannomas shows areas of cellularity and areas of myxoid matrix, as also observed in conventional schwannomas. There is, however, a relative loss of cellular regions, which tend to be fibrosed or sclerotic. These areas may degenerate into hematomas and cysts, leading to an overall decreased densitity. Nuclear palisades, seen in classic schwannomas, are absent and large intra-nuclear invaginations are characteristically present [7]. The degenerative histological features of ancient schwannomas are evident in their radiographic features as well-circumscribed complex cystic masses with inhomogeneous contrast enhancement as noted in the cases presented. Non-enhancing areas on CT imaging correspond to regions of cystic degeneration, with contrast enhancement seen in surrounding tissues [5]. MRI with gadolinium enhancement has been advocated as superior to CT in demonstrating tumor cystic degeneration, defining margins and in some cases identifying the point of neuronal origin [20,21]. However, radiographic modalities do not differentiate benign from malignant disease unless tumor invasion or metastasis is seen. Increased accumulation of 2-deoxy-[(18)F] fluoro-D-glucose (FDG) on PET scanning has been previously reported in cases of schwannomas, and was noted in one case in our series [22]. The role of PET in assessing the malignant potential of schwannomas is however undetermined. Surgery is usually required for definitive diagnosis of these tumors and differentiation from other retroperitoneal malignancies. Tumor enucleation, with preservation of vital structures in the vicinity, is the preferred surgical approach when a diagnosis of retroperitoneal schwannoma is highly suspected, since these tumors have not been reported to recur following excision.

We described three cases of retroperitoneal ancient schwannomas, all with features concerning for malignancy and treated by radical excision. All three patients were symptomatic, with two presenting with back and leg pain and associated weakness. Malignant transformation of retroperitoneal schwannomas appears to extremely rare. If a diagnosis of ancient schwannoma is entertained based on imaging and histology, then consideration for less radical surgery may be appropriate in selected cases.

## Consent

Institutional review board approval was obtained to conduct to the case reviews. A copy of the approval documents is available for review by the Editor-in-Chief of this journal.

## Competing interests

The authors declare that they have no competing interests.

## Authors' contributions

HAC, MN, NJC and ETC collected the patient details. HAC and MN reviewed the literature. HAC wrote the paper with the assistance of MN, ETK, NJS, RC and KFS. JL examined the pathology specimens and provided histology details included in the manuscript. All authors were involved in the editing and reviewing of the initial document. KFS, MN, RC, ETC and NJG were involved in the study conception and design. All authors read and approved the final manuscript.
